# A Conditioning Sciatic Nerve Lesion Triggers a Pro-regenerative State in Primary Sensory Neurons Also of Dorsal Root Ganglia Non-associated With the Damaged Nerve

**DOI:** 10.3389/fncel.2019.00011

**Published:** 2019-02-04

**Authors:** Petr Dubový, Ilona Klusáková, Ivana Hradilová-Svíženská, Václav Brázda, Marcela Kohoutková, Marek Joukal

**Affiliations:** Department of Anatomy, Laboratory of Cellular and Molecular Neurobiology, Faculty of Medicine, Masaryk University, Brno, Czechia

**Keywords:** unilateral nerve injury, primary sensory neurons, pro-regenerative state, GAP-43, SCG-10, IL-6, ulnar nerve crush, neurite outgrowth assay

## Abstract

The primary sensory neurons of dorsal root ganglia (DRG) are a very useful model to study the neuronal regenerative program that is a prerequisite for successful axon regeneration after peripheral nerve injury. Seven days after a unilateral sciatic nerve injury by compression or transection, we detected a bilateral increase in growth-associated protein-43 (GAP-43) and superior cervical ganglion-10 (SCG-10) mRNA and protein levels not only in DRG neurons of lumbar spinal cord segments (L4-L5) associated with injured nerve, but also in remote cervical segments (C6-C8). The increase in regeneration-associated proteins in the cervical DRG neurons was associated with the greater length of regenerated axons 1 day after ulnar nerve crush following prior sciatic nerve injury as compared to controls with only ulnar nerve crush. The increased axonal regeneration capacity of cervical DRG neurons after a prior conditioning sciatic nerve lesion was confirmed by neurite outgrowth assay of *in vitro* cultivated DRG neurons. Intrathecal injection of IL-6 or a JAK2 inhibitor (AG490) revealed a role for the IL-6 signaling pathway in activating the pro-regenerative state in remote DRG neurons. Our results suggest that the pro-regenerative state induced in the DRG neurons non-associated with the injured nerve reflects a systemic reaction of these neurons to unilateral sciatic nerve injury.

## Introduction

It is well-known that besides extrinsic factors, activation of a neuronal regenerative program is necessary for successful axon regeneration after peripheral nerve injury. The primary sensory neurons, whose bodies are in the dorsal root ganglia (DRG), are a useful model to study the mechanisms regulating the neuronal regeneration program after axotomy. The DRG neurons have peripheral and central branches of afferent axons with different responses to injury. Injury to peripheral axonal branches induces transcription-dependent changes of regeneration-associated genes and proteins that promote axon regeneration by enhancing the regeneration potential of DRG neurons (Liu et al., 2011). In contrast, central axonal branches extending into the dorsal columns of the spinal cord fail to regenerate when injured because insufficient activation of the neuronal regeneration program (Schwaiger et al., 2000; Qiu et al., 2005). However, a conditioning lesion of the peripheral nerve, where peripheral axonal branches are injured beforehand, triggers a regenerative program in DRG neurons that is sufficient to allow regeneration also of central axonal branches (Neumann and Woolf, 1999). This phenomenon of a conditioning peripheral nerve lesion with the activation of the pro-regenerative state in DRG neurons is at least partly associated with upregulation of some neuropoietic cytokines including IL-6 (Cafferty et al., 2001; Zigmond, 2011, 2012). It was also shown that increased axon regeneration was conditioned in the homologous nerve contralateral to the injured nerve (Yamaguchi et al., 1999; Ryoke et al., 2000).

In our previous experiments we have found bilateral increased levels of IL6, as well as its receptor mRNA and protein, not only in DRG associated with the injured sciatic nerve, but also in remote cervical DRG (Brázda et al., 2013; Dubový et al., 2013). Moreover, unilateral sciatic nerve injury induced bilateral activation of STAT3 by phosphorylation at the tyrosine-705 (Y705) position in DRG neurons of both lumbar and cervical segments (Dubový et al., 2018a) that is a critical transforming factor of the neuronal pro-regenerative state (Bareyre et al., 2011; Zigmond, 2011). Based on our own and other previously published results, we hypothesize that nerve injury may stimulate the pro-regenerative state not only in the DRG neurons associated with damaged axons, but also in DRG neurons not directly associated with the injured nerve.

Activation of the neuronal pro-regenerative state is characterized by upregulation of various transforming factors, regeneration-associated genes and proteins which are important intrinsic determinants of neuronal regeneration capacity (Mar et al., 2014; Rishal and Fainzilber, 2014; Chandran et al., 2016). For example, phosphorylation and activation of the transcription factor cJun (Itoh et al., 2009; Frey et al., 2015) and the mitogen-activated protein kinase p38 (p38 MAPK; Verma et al., 2005; Nix et al., 2011) is associated with induction of the pro-regenerative state in DRG neurons following nerve injury.

Regeneration-associated proteins like growth-associated protein-43 (GAP-43) or superior cervical ganglion-10 (SCG-10) are generally used as markers for detecting the neuronal pro-regenerative state (Bonilla et al., 2002; Mason et al., 2002). GAP-43 is the prototypical GAP, is expressed at high levels in neurons during development and concentrated in the axonal growth cone (Skene and Willard, 1981). DRG neurons of naïve rats display a low basal level of GAP-43 that is significantly increased after nerve injury (Stewart et al., 1992; Schreyer and Skene, 1993; Liabotis and Schreyer, 1995). The SCG-10 protein, also known as stathmin 2, is a neuron-specific member of the stathmin family (Sugiura and Mori, 1995) that is upregulated specifically in primary sensory neurons regenerating their axons (Shin et al., 2014).

The goal of the present study was to investigate whether a sciatic nerve injury can activate the pro-regenerative state of DRG neurons not only in the corresponding lumbar but also in remote cervical spinal cord segments. The pro-regenerative state of DRG neurons was shown by the upregulation of GAP-43 and SCG-10 mRNAs and proteins as well as activation of cJun and p38 in correlation with the extent of regenerated axons after ulnar nerve crush following prior sciatic nerve injury. Increased axon regeneration capacity in cervical DRG neurons initiated by the conditioning sciatic nerve lesion was also confirmed by neurite outgrowth assay of *in vitro* cultivated DRG neurons. Moreover, intrathecal injection of IL-6 revealed that this cytokine can mediate this systemic reaction of DRG along the neuroaxis after unilateral sciatic nerve lesion.

## Materials and Methods

### Animals and Surgical Treatment

The experiments were performed in 194 adult male rats (Wistar, 250–280 g, Anlab, Brno, Czechia) housed in 12 h light/dark cycles at a temperature of 22–24°C under specific pathogen-free conditions in the animal housing facility of Masaryk University. Sterilized standard rodent food and water were available *ad libitum*. Animals for surgical treatments were anesthetized using a mixture of ketamine (40 mg/ml) and xylazine (4 mg/ml) administered intraperitoneally (0.2 ml/100 g body weight). All surgical procedures were carried out under sterile conditions by the same person according to protocols approved by the Animal Investigation Committee of the Faculty of Medicine, Brno, Czechia.

The right sciatic nerve of rats was exposed in mid-thigh, ligated with two ligatures and cut (complete sciatic nerve transection, CSNT). The proximal nerve stump was fixed in muscles to protect the distal stump from reinnervation. A longitudinally slit silicone tube of 2-mm length and 1 mm internal diameter was placed around the right sciatic nerve of rats to reduce the nerve diameter (sciatic nerve compression, SNC; Schmid et al., 2013). The tube was tied in place with a sterile thread to close and prevent tube from opening. The muscles and skin were closed with 5/0 sutures. The right sciatic nerve of sham-operated rats was carefully exposed without any lesion.

**Figure 1:**
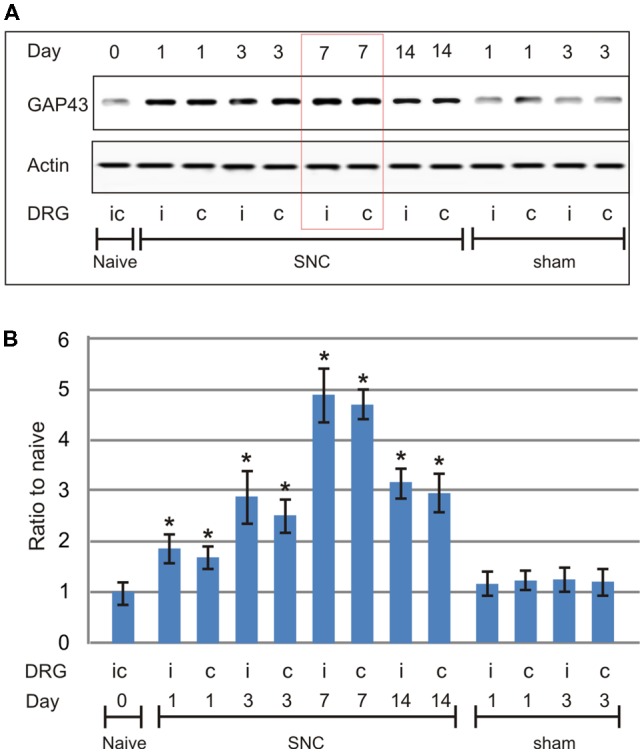
Results of western blot analysis of growth-associated protein-43 (GAP-43) protein levels in cervical dorsal root ganglia (DRG; C6-C8) removed from naïve rats as well as sham- and sciatic nerve compression (SNC)-operated rats. Sham-operated rats were left to survive for 1 and 3 days, SNC-operated rats for 1, 3, 7 and 14 days (*n* = 3 for each group). Upper panel **(A)** shows a representative western blot with GAP-43 protein in cervical DRG ipsilateral (i) and contralateral (c) to unilateral SNC. Equal loading of proteins was confirmed by actin levels (Actin). Lower panel **(B)** shows densitometry of the individual protein bands after normalization to actin; the intensities of the bands from naïve cervical DRG were as arbitrarily set to 1. *Significant difference (*p* < 0.05) compared to naïve or sham-operated rats in a Mann-Whitney U-test.

To determine the progression in time of the pro-regenerative state in cervical DRG after sciatic nerve lesion, a set of SNC-operated rats from the pilot study was left to survive for 1, 3, 7, and 14 days and compared with naïve rats or sham-operated rats surviving for 1 and 3 days (*n* = 3 for each group). Based on the pilot study in which GAP-43 peaked at 7 days after unilateral sciatic nerve lesion (Figure 1), further groups of operated and sham-operated rats were left to survive for 7 days after surgical treatment and used for bilateral analysis of the neuronal regeneration program in both lumbar and cervical DRG.

### Quantitative Immunohistochemical Analysis

Naïve rats and sham-, SNC- and CSNT-operated rats (*n* = 6 for each group) were deeply anesthetized with a lethal dose of sodium pentobarbital (70 mg/kg body weight, i.p.) and perfused transcardially with 500 ml phosphate-buffered saline (PBS, pH 7.4) followed by 500 ml of Zamboni’s fixative (Zamboni and Demartin, 1967). The L4-L5 and C6-C8 DRG from both sides were detected in their intervertebral foramina following total laminectomy and foraminotomy. The DRG were removed, immersed separately in Zamboni’s fixative at 4°C overnight, and then collected separately into samples of ipsilateral (L-DRGi) and contralateral (L-DRGc) lumbar as well as ipsilateral (C-DRGi) and contralateral (C-DRGc) cervical DRG for each group of rats (naïve, sham-, SNC-, and CSNT-operated).

The DRG samples were washed in 20% phosphate-buffered sucrose for 12 h, blocked in Tissue-Tek^®^ OCT compound (Miles, Elkhart, IN, USA) and cut to prepare serial longitudinal cryostat sections (12 μm). The DRG sections were mounted on chrome-alum covered slides and processed for indirect immunohistochemical staining for GAP-43 and SCG-10. Briefly, DRG sections of lumbar and cervical segments of naïve, sham-, SNC- and CSNT-operated rats were immunostained simultaneously under the same conditions. Sections were washed with PBS containing 0.05% Tween 20 (PBS-T) and 1% bovine serum albumin (BSA) for 10 min, treated with 5% normal donkey serum in PBS-T for 30 min, then incubated with 25 μl of mouse monoclonal antibody against GAP-43 (1:500; Sigma, Ronkonkoma, NY, USA) and rabbit polyclonal antibody against SCG-10 (1:1,000; LSBio, Seattle, WA, USA), phospho-cJun (1:100; Cell Signaling, New York, NY, USA) or phospho-p38 (1:200; Chemicon Int., Temecula, CA, USA) in a humid chamber at room temperature (21–23°C) for 12 h. The immunohistochemical reaction was visualized by treatment with FITC-conjugated and affinity-purified donkey anti-mouse or anti-rabbit secondary antibody (1:100; Millipore, USA) for 90 min at room temperature. The control sections were incubated without the primary antibody. Sections were stained with Hoechst 33342 to detect cell nuclei, mounted in aqueous mounting medium (Vectashield; Vector Laboratories, Burlingame, CA, USA) and analyzed using an epifluorescence microscope (Nikon Eclipse, Nikon, Czechia) equipped with a camera (DFC-480; Leica Microsystems) and a stabilized power supply for the lamp housing.

The neuronal diameter, GAP-43 and SCG-10 immunofluorescence intensities were measured using a NIS-Elements image analysis system (Nikon, Czechia) as previously (Dubový et al., 2002, 2013). At least 100 neuronal profiles containing nuclei were measured for each animal group. The sizes of DRG neurons in sections for immunofluorescence were categorized as small (<25 μm), medium (25–40 μm), and large (>40 μm) according to their diameters calculated from the areas of neuronal profiles. The immunofluorescence intensities were expressed as mean intensity ± SD.

### Western Blot Analysis

In the pilot study, the cervical DRG (C6-C8) of naïve rats and rats surviving 1 and 3 days after sham-operation and 1, 3, 7 and 14 days after SNC-operation (*n* = 3 for each group) were removed bilaterally, flash-frozen in liquid nitrogen and stored at −80°C until western blot analysis of GAP-43.

The results of GAP-43 and SCG-10 levels obtained by quantitative immunohistochemistry in DRG neurons 7 days after sciatic nerve lesions were verified by western blot analysis. The DRG from lumbar (L4-L5) and cervical (C6-C8) levels (*n* = 3 for each group in three independent experiments) were collected as described under aseptic conditions. Samples of ipsilateral (L-DRGi) and contralateral (L-DRGc) lumbar DRG as well as ipsilateral (C-DRGi) and contralateral (C-DRGc) cervical DRG from each group of rats were flash-frozen in liquid nitrogen and stored at −80°C until analysis.

The DRG samples were homogenized in PBS containing protease inhibitors (LaRoche, Switzerland) with 0.1% Triton X-100, and centrifuged at 10,000 *g* for 5 min at 4°C. Proteins were separated by SDS-polyacrylamide gel electrophoresis (Brazda et al., 2006) and transferred to nitrocellulose membranes by electroblotting (BioRad). Blots were blocked using 1% BSA in PBST (3.2 mM Na_2_HPO_4_, 0.5 mM KH_2_PO_4_, 1.3 mM KCl, 135 mM NaCl, 0.05% Tween 20, pH 7.4) for 1 h and incubated with anti-GAP-43 mouse monoclonal (1:1,000; Sigma, Ronkonkoma, NY, USA), rabbit polyclonal anti-phosphorylated S41-GAP43 (1:500; Thermo Fisher Scientific, Waltham, MA, USA) or anti-SCG-10 (1:500; LSBio, Seattle, WA, USA) antibodies overnight. Blots were washed in PBST and incubated with peroxidase-conjugated anti-mouse or anti-rabbit IgG (1:1,000; Sigma, Seattle, WA, USA) at room temperature for 1 h. Protein bands were visualized by the ECL detection kit (Amersham, USA) on the chemiluminometer reader LAS-3000 (Fuji, Japan) and analyzed using densitometry image software. The levels of proteins were normalized to the value of naïve DRG, which was arbitrarily set as one.

### Real Time RT-PCR

The expression of GAP-43 and SCG-10 mRNAs in DRG was analyzed by real-time PCR (RT-PCR). Whole DRG from each group of rats (*n* = 3 for each group in three independent experiments) were removed under aseptic conditions from lumbar (L4-L5) and cervical (C6-C8) segments of both sides, collected as ipsilateral and contralateral samples and stored in RNA later (Thermo Fisher Scientific, Inc., Waltham, MA, USA) at 4°C. First-strand synthesis was performed using TaqMan^®^ High Capacity RNA-to-cDNA Kit and the quality and concentration evaluated by optical density using NanoDrop. PCR amplification, in triplicate for each sample, was performed using ABI Prism 7300, TaqMan^®^ Gene Expression Master Mix, and TaqMan^®^ Gene Expression Assay Probes FAM™ (Thermo Fisher Scientific, Inc., Waltham, MA, USA) for the target genes GAP-43 (assay ID-Rn01474579_m1) and SCG-10 (assay ID—Rn00584886_m1). Determinations were made with reference to the reporter gene encoding rat actin (actin, beta—Rn00667869_m1) Endogenous Control (VIC^®^). The polymerase activation step at 95°C for 15 min was followed by 40 cycles of 15 s at 95°C and 60 s at 60°C. The validity of the results was checked by running appropriate negative controls (water instead of cDNA by for PCR amplification; omitting reverse transcriptase for cDNA synthesis). Specific mRNA levels were calculated after normalizing to actin mRNA in each sample. Relative expression was determined using the Comparative Ct Model (ΔΔCt) with actin as the housekeeping gene. Data were presented as relative mRNA units compared with control values (expressed as fold over naive value).

### *In vivo* Assay of Axon Regeneration in Crushed Ulnar Nerve

Rats with prior operation on SNC (*n* = 6) or CSNT (*n* = 6) for 7 days were re-operated to expose and mobilize a short segment of the right ulnar nerve. The right ulnar nerve was also mobilized in a control group without any previous sciatic nerve injury (*n* = 6). The ulnar nerve was then crushed using a clamp of a defined force of 1.9 N for 2 × 1 min (Ronchi et al., 2009) under a stereoscopic microscope. The distal margin of the crush injury was indicated with a 10-0 epineurial suture and the skin wound was closed with 5/0 sutures.

To investigate peripheral axon regeneration, the ulnar nerves were removed 1 day after the crush injury following pericardial infusion with Zamboni fixative solution and ulnar nerve samples were fixed by immersion in Zamboni fixative solution overnight. After washing with 10% sucrose in PBS, longitudinal cryostat sections of 10 μm thickness were cut and immunostained for SCG-10, which is a more selective marker of regenerating sensory axons than GAP-43 (Shin et al., 2014). SCG-10 fluorescence intensity was analyzed along the length of the nerve distal to the crush; the regeneration index was determined by measuring the length of the longest SCG-10 decorated axons from the crush site (Abe et al., 2010). The length of SCG-10+ axons was measured by a person blind to the experimental conditions in every third section using a NIS-Elements image analysis system (Nikon, Czechia).

### *In vitro* Assay of Increased Axonal Outgrowth of Cervical DRG Neurons Induced by Sciatic Nerve Lesion

An *in vitro* culture of dissociated DRG neurons was prepared according to a modified protocol (Christie et al., 2010). Sham-operated rats (*n* = 4) and rats operated by SNC (*n* = 4) or CSNT (*n* = 4) for 7 days were deeply anesthetized by an intraperitoneal application of sodium pentobarbital (70 mg/kg) and killed by decapitation. The DRG of lumbar (L4-L5) and cervical (C6-C8) segments were removed bilaterally under aseptic conditions following laminectomy. The samples were collected in ice-cold Ca^2+^/Mg^2+^-free Hank’s buffered salt solution (CMF-HBSS, Sigma Aldrich), and the spinal roots and connective tissues were removed.

DRG were then dissociated by incubation in medium containing 0.1% collagenase type I (5,000 U/ml) for 90 min followed by 0.25% trypsin/EDTA at 37°C for 25 min. The DRG suspension was prepared by triturating through glass pipette tips, washed twice with Dulbecco’s Modified Eagle’s Medium/Nutrient F-12 Ham (DMEM/F12) supplemented with 10% fetal bovine serum (FBS; all from Sigma Aldrich) and the suspension was spun for 5 min at 1,500 rpm at 4°C. The cells were resuspended and placed into a culture medium of DMEM/F12 supplemented with 2 mM glutamine, 100 U/ml Penicillin and 100 μg/ml Streptomycin (all from Sigma Aldrich), N2 and B27 (ThermoFisher Sci., diluted according to the manufacturer’s instructions). The cells were re seeded at a density of 200 cells on glass cover slips previously coated with Geltrex^®^ (ThermoFisher Sci.).

Cultures were incubated at 37°C in a humidified atmosphere containing 5% CO_2_ for 2 days, washed in PBS, fixed by Zamboni solution for 20 min and immunostained with mouse anti-β tubulin III primary antibody (Sigma, 1:500) overnight at 4°C. The cells were then washed in PBS followed by incubation with goat anti-mouse TRITC-conjugated secondary antibody (Life Technologies, 1:1,000) for 90 min. Finally, cells were washed in PBS, stained with Hoechst 33342 to detect cell nuclei and mounted in a Vectashield aqueous mounting medium (Vector Laboratories, Burlingame, CA, USA). Three slides for each experimental group were analyzed under a Nikon Eclipse NI-E epifluorescence microscope equipped with a Nikon DS-Ri1 camera (Nikon, Prague, Czechia) using a 10× objective by a person blind to the experimental conditions.

To analyze neurite outgrowth and length, digital images of at least 100 randomly selected nucleate neurons per cover slip were acquired. Neurites longer than the diameter of the neuronal bodies were analyzed in digital pictures converted to grayscale for better visualization. Neurite outgrowth initiation was quantified as the number of neurites per neuron counted using the Count and Taxonomy module of NIS- Elements software (Nikon, Prague, Czechia). Axonal elongation was analyzed by taking the total length of neurites per neuron when axons of individual neurons were traced and measured using the Neurite Tracer Plugin for ImageJ (Pool et al., 2008). The mean number of neurites per neuron and total neurite length per neuron were calculated from triplicate experiments and data were present as mean ± SD.

### *In vivo* Assay of Axon Regeneration in the Crushed Ulnar Nerve and Changes in the Pro-regenerative State of Cervical DRG Neurons After Intrathecal Injection of IL-6 or JAK2 Inhibitor AG490

Recombinant rat IL-6 protein (R&D Systems) was dissolved in artificial cerebrospinal fluid (ACSF; Hylden and Wilcox, 1980) at 20 ng/10 μl. AG490 (Sigma), an inhibitor of JAK2, was dissolved in ACSF at a concentration of 5 μM.

A solution of IL-6 (10 μl) or ACSF (10 μl) along with a further 10 μl ACSF was injected via a micro syringe into the lumbar subarachnoid space of intact rats (*n* = 2 for each group). Animals were left to survive for 1 day and cervical DRG (C6-C8) were removed following pericardial infusion with Zamboni fixative solution. Longitudinal DRG sections were double immunostained with mouse monoclonal antibody against GAP-43 (1:500; Sigma, USA) and rabbit polyclonal anti-STAT3 (Y705) antibody (1:100; Santa Cruz, CA, USA). The immunofluorescence reaction was visualized by treatment with FITC-conjugated donkey anti-mouse and TRITC-conjugated donkey anti-rabbit secondary antibodies (1:100; Millipore, USA) for 90 min at room temperature. Activation and nuclear translocation of STAT3 as well as GAP-43 immunofluorescence intensity were measured using a NIS-Elements image analysis system (Nikon, Czechia) as described previously.

To investigate *in vivo* the role of IL-6 in triggering the pro-regenerative state in cervical DRG, the right ulnar nerve was crushed as described (*n* = 8). A solution of IL-6 (10 μl) or ACSF (10 μl) along with a further 10 μl ACSF was then injected via a micro syringe into the lumbar subarachnoid space (*n* = 4 for each group). The ulnar nerves were removed 1 day later (after the crush and intrathecal administration of IL-6) and fixed with Zamboni fixative solution. Axon regeneration was assessed on longitudinal cryostat sections (10 μm thick) immunostained for SCG-10 and analyzed as described.

To test whether the JAK2/STAT3 signaling pathway is involved in inducing the pro-regenerative state of cervical DRG neurons after prior sciatic nerve injury, the right sciatic nerve of 8 rats was cut (CSNT). After 7 days, the rats with prior CSNT were re-operated to expose and crush the right ulnar nerve. 10 μl of AG490 solution (5 μM) or ACSF (10 μl), along with a further 10 μl ACSF was then injected via a micro syringe into the subarachnoid space of the cisterna magna (*n* = 4 for each group). The length of regenerated SCG10+ axons was assessed on longitudinal cryostat sections (10 μm thick) 1 day after the ulnar nerve crush and intrathecal injection of ACSF or AG490 as described above. In addition, cryostat sections of cervical DRG (C6-C8) ipsilateral to the ulnar nerve crush were double immunostained for GAP-43 and STAT3 and analyzed using a NIS-Elements image analysis system (Nikon, Czechia) as described above.

### Enzyme-Linked Immunosorbent Assay (ELISA) of IL-6 in Rat Plasma

Three naïve rats and those operated on to create SNC or CSNT for 1, 3, and 7 days (*n* = 3 for each group), as well as sham-operated rats for 3 (*n* = 3) and 7 (*n* = 3) days were sacrificed by CO_2_ inhalation. Blood samples were obtained by intracardiac puncture and collected into tubes containing heparin and protease inhibitor cocktail (LaRoche, Switzerland). Plasma was separated by centrifugation (2,500 *g* for 12 min) and stored at −60°C until analyzed. Total protein was measured by Nanodrop ND-1000 (Thermo Scientific) and the level of IL-6 protein was assessed using an ELISA kit with a sensitivity of 5 pg/ml (BioSource International, USA) according to the manufacturer’s instructions. Measurement was carried out on a SUNRISE Basic microplate reader (Tecan, Salzburg, Austria) and the data were normalized as picograms of IL-6 protein to 100 μg of total protein. IL-6 protein levels were compared to baseline values in plasma from naïve rats, which was arbitrarily set as one. Data were expressed as mean ± SD.

### Statistical Analyses

Statistical differences between data of immunofluorescence intensities, western blot analysis and RT-PCR of naïve DRG neurons and DRG neurons of sham-operated rats or rats with SNC and CSNT were tested using a Mann-Whitney U-test (*p* < 0.05). The same statistical analysis was used to compare IL-6 protein levels in plasma of naïve and sham-operated or SNC- and CSNT-operated rats. The mean number of neurites per neuron and total neurite length per neuron were compared between DRG neurons of sham- and SNC- or CSNT-operated rats using one-way ANOVA and Tukey’s *post hoc* test to determine statistical significance. All statistical analyses were performed using STATISTICA 9.0 software (StatSoft, Inc., Tulsa, OK, USA).

## Results

### Immunohistochemical and Western Blot Analysis of GAP-43 and SCG-10 Proteins

Western blot analysis of GAP-43 in cervical DRG of rats surviving 1, 3, 7, and 14 days after SNC showed the protein level peaked at 7 days with a drop at 14 days when compared to cervical DRG of naïve or sham-operated rats (Figure 1). Therefore, subsequent analyses illustrating the pro-regenerative state of cervical DRG neurons after sciatic nerve lesions were performed in rats 7 days after SNC or CSNT.

**Figure 2:**
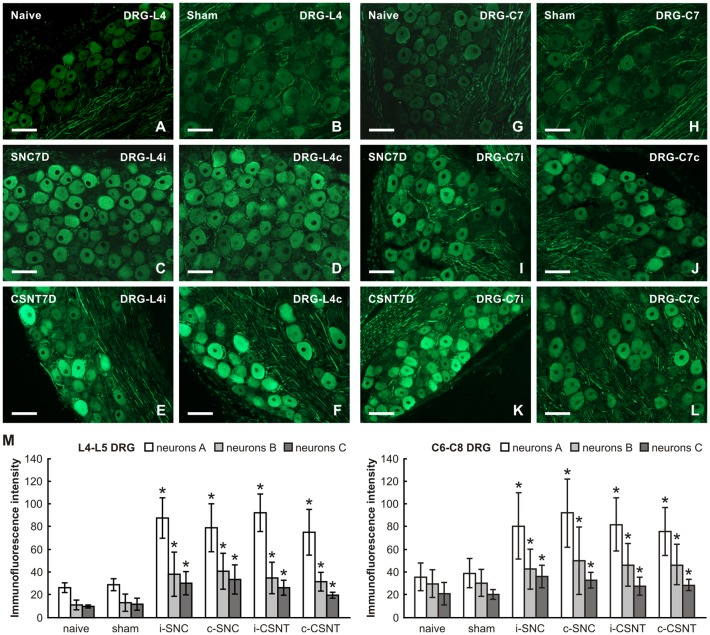
Representative pictures of cryostat sections through the DRG from naïve **(A,G)**, sham-operated **(B,H)** rats and rats with unilateral sciatic nerve compression (SNC; **C,D,I,J**) or complete sciatic nerve transection (CSNT; **E,F,K,L**) for 7 days. The sections of ipsilateral **(C,E)** and contralateral **(D,F)** DRG of the L4 spinal segment as well as ipsilateral **(I,K)** and contralateral **(J,L)** DRG of the C7 spinal segment were incubated under the same conditions with mouse monoclonal antibody recognizing GAP-43. Scale bars = 75 μm. **(M)** Graph illustrating the mean intensity of GAP-43 immunofluorescence in individual DRG neuron-size classes of cervical (C6-C8) and lumbar (L4-L5) spinal segments ipsilateral (i) and contralateral (c) to unilateral SNC and transection (CSNT) for 7 days; *n* = 6 for each group. Neurons A ≥ 40 μm; Neurons B 25–40 μm; Neurons C ≤ 25 μm. *Significant difference (*p* < 0.05) compared to naive or sham-operated rats in a Mann-Whitney U-test.

The lumbar and cervical DRG neurons of naïve and sham-operated rats displayed a low basal level of GAP-43 immunostaining. GAP-43 immunofluorescence increased strongly in large and medium-sized neurons of both ipsilateral and contralateral lumbar DRG 7 days after SNC and CSNT. Some medium- and small-sized neurons displayed only weak GAP-43 immunopositivity. Sections of cervical DRG also demonstrated a similar pattern of bilaterally increased GAP-43 immunofluorescence (Figure 2).

Image analysis revealed a bilateral increase of mean GAP-43 immunofluorescence intensity in all size-classes of neurons in both lumbar and cervical DRG after unilateral SNC and CSNT in comparison with naïve and sham-operated animals. Sciatic nerve injury induced a greater increase in GAP-43 immunofluorescence intensity in large neurons than in medium- and small-sized neurons of both lumbar and cervical DRG. Further, a more significant increase in GAP-43 immunofluorescence was measured bilaterally in lumbar (3.3–2.6 times) than in cervical DRG neurons (2.5–1.9 times) compared to naïve or sham-operated rats. However, no significant differences were found between individual size-classes of neurons in the SNC and CSNT experimental groups (Figure 2M).

**Figure 3:**
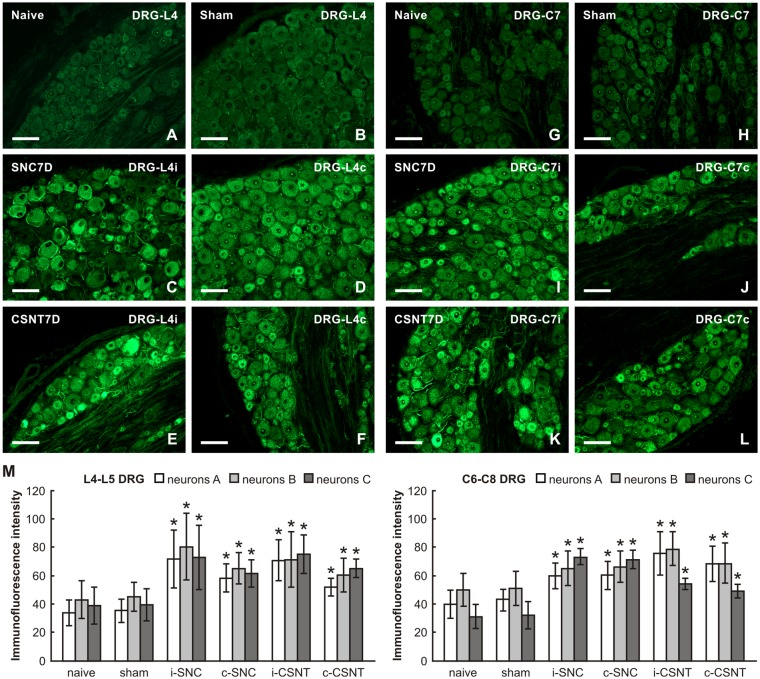
Representative pictures of cryostat sections through the DRG from naïve **(A,G)**, sham-operated **(B,H)** rats and rats with unilateral SNC **(C,D,I,J)** or CSNT **(E,F,K,L**) for 7 days. The sections of ipsilateral **(C,E)** and contralateral **(D,F)** DRG of the L4 spinal segment as well as ipsilateral **(I,K)** and contralateral **(J,L)** DRG of the C7 spinal segment were incubated under the same conditions with rabbit polyclonal antibody recognizing superior cervical ganglion-10 (SCG-10). Scale bars = 75 μm. **(M)** Graph illustrating the mean intensity of SCG-10 immunofluorescence measured in individual DRG neuron-size classes of cervical (C6-C8) and lumbar (L4-L5) spinal segments ipsilateral (i) and contralateral (c) to unilateral SNC and transection (CSNT) for 7 days; *n* = 6 for each group. Neurons A ≥ 40 μm; Neurons B 25–40 μm; Neurons C ≤ 25 μm. *Significant difference (*p* < 0.05) compared to naive or sham-operated rats in a Mann-Whitney U-test.

Basal SCG-10 immunofluorescence was very low or absent in the lumbar and cervical DRG neurons of naïve rats. Most neurons of all sizes in lumbar and cervical DRG of both sides displayed significantly enhanced SCG-10 immunofluorescence intensity 7 days after SNC and CSNT compared to DRG from naïve or sham-operated rats. However, the increase in SCG-10 immunofluorescence intensity was lower than for GAP-43 (approximately 1.8–2 times in lumbar and 1.4–2.3 times in cervical DRG; Figures 3A–L). Moreover, image analysis of SCG-10 immunofluorescence intensity did not show any preferential increase among individual size-classes of DRG neurons (Figure 3M).

**Figure 4:**
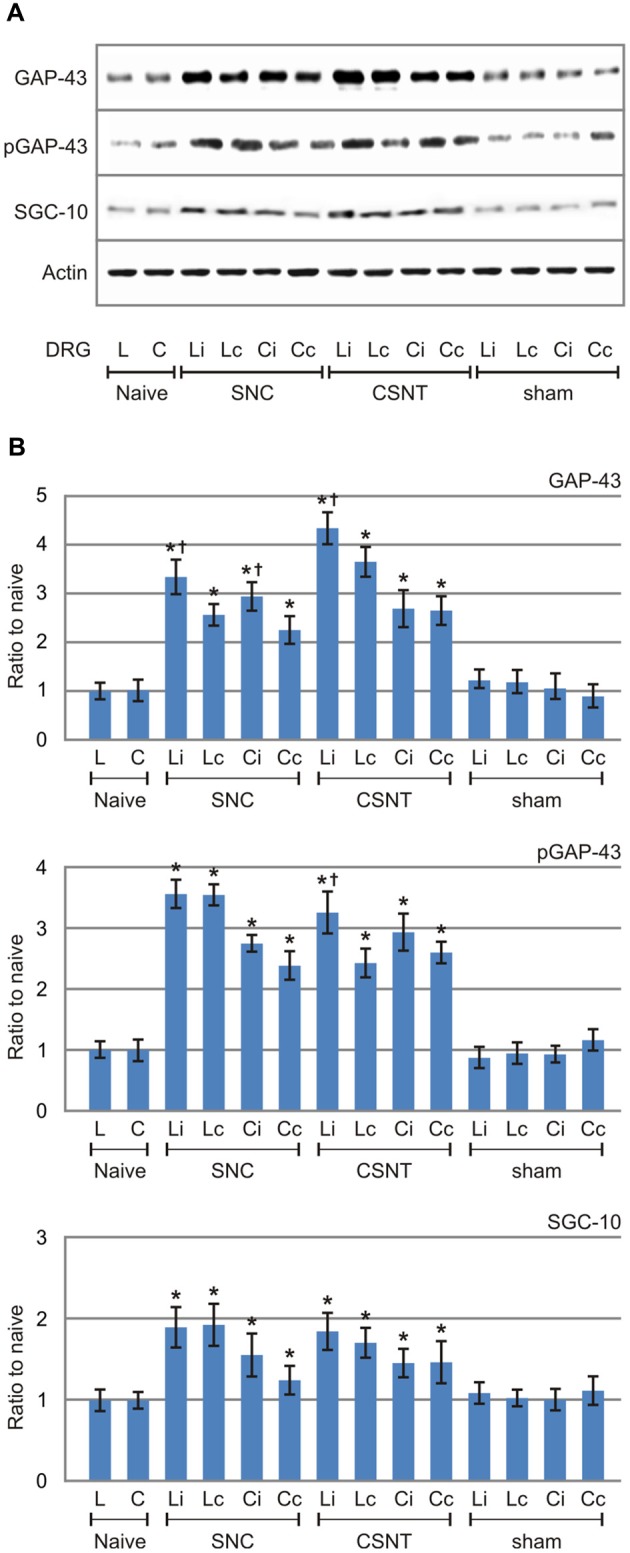
Results of western blot analysis of GAP-43, pGAP-43 and SCG-10 protein levels in DRG of L4-L5 (L) and C6-C8 (C) segments removed from ipsilateral (i) and contralateral (c) sides of naïve as well as sham-, SNC- and CSNT-operated rats for 7 days. Upper panel **(A)** illustrates representative western blots of DRG from three rats for each group. Equal loading of proteins was confirmed by actin levels (Actin). The same Actin controls were used for analysis of GAP-43, pGAP-43 and SCG-10 protein levels in this set representative western blots. Lower panels **(B)** show densitometry of individual protein bands after normalization to actin from three independent experiments; the intensities of the bands from naïve DRG were as arbitrarily set to 1. *Significant difference (*p* < 0.05) when compared to sham-operated rats; ^†^Significant difference (*p* < 0.05) compared to contralateral DRG in a Mann-Whitney U-test.

The increased levels of GAP-43 and SCG-10 proteins induced by SNC and CSNT and detected in the lumbar and cervical DRG of both sides by quantitative immunohistochemistry were verified by western blot analysis. Total GAP-43 and SCG-10 proteins were also significantly increased bilaterally in both lumbar and cervical DRG after unilateral SNC and CSNT for 7 days compared to naïve or sham-operated controls. Seven days after SNC or CSNT, the levels of GAP-43 protein were increased bilaterally in the lumbar and cervical DRG about two-to-three times compared to naïve and sham-operated controls. As was expected, GAP-43 protein levels shot up significantly in lumbar DRG after CSNT than SNC. Levels of GAP-43 protein in cervical DRG also increased significantly compared to controls, but the elevation was not to the extent seen in lumbar DRG (Figures 4A,B).

GAP-43 is activated by protein kinase C-mediated phosphorylation at serine 41 (S41) that promotes the polymerization and stabilization of filamentous actin (F-actin) related to axon growth and sprouting (Tsai et al., 2007). Although in our experiments we used an antibody recognizing both unphosphorylated and phosphorylated GAP-43, the western blot analysis verified levels of activated GAP-43 using a specific antibody against phosphorylated GAP-43 (pGAP-43). The results demonstrated that activated pGAP-43 was increased bilaterally not only in lumbar but also in cervical DRG after SNC or CSNT compared to naïve or sham-operated controls. The magnitude of pGAP-43 increase was very similar to the increased GAP-43 levels. However, in contrast to significantly higher GAP-43 protein levels in ipsilateral than contralateral lumbar DRG after both SNC and CSNT, pGAP-43 protein level was significantly higher only in ipsilateral lumbar DRG after CSNT (Figures 4A,B).

In contrast to GAP-43 and pGAP-43, the increase in levels of SCG-10 was less marked (about 1.5–1.8-fold) and the increase was more or less the same on both sides. Moreover, no significant differences in SCG-10 levels were found in lumbar DRG from SNC- and CSNT-operated animals. Similar protein levels of SCG-10 were also measured in cervical DRG after SNC and CSNT, as in the case of GAP-43 and pGAP-43 (Figures 4A,B).

### RT-PCR Analysis of GAP-43 and SCG-10 mRNA

RT PCR was used to determine the levels of the relevant GAP-43 and SCG-10 mRNA in cervical and lumbar DRG 7 days after unilateral sciatic nerve injury by SNC or CSNT. Samples of cervical and lumbar DRG from sham-operated animals displayed no significant changes in SCG-10 and GAP-43 mRNA levels compared to naïve controls.

Levels of GAP-43 and SCG-10 mRNAs were significantly increased bilaterally in both cervical and lumbar DRG 1 week following sciatic nerve injury by SNC or CSNT compared to naïve or sham-operated controls. GAP-43 mRNA increased bilaterally in lumbar DRG to a higher level after CSNT than SNC. However, a statistically significant difference between ipsilateral and contralateral lumbar DRG was found only in the CSNT group. In contrast to lumbar DRG, cervical ones displayed a bilateral increase of GAP-43 mRNA that was greater after SNC than after CSNT.

**Figure 5:**
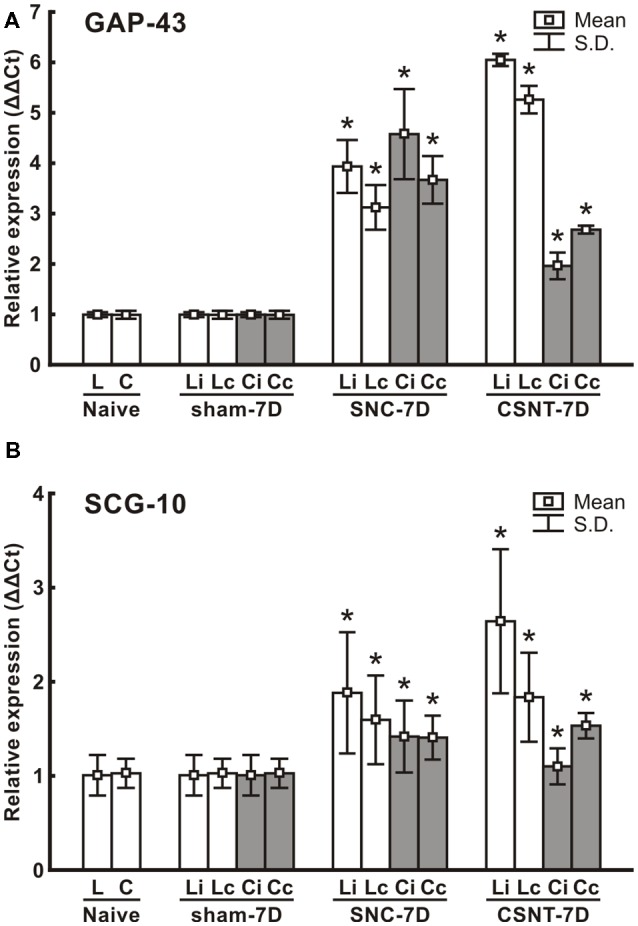
Results of real-time PCR (RT-PCR) of relative GAP-43 **(A)** and SCG-10 **(B)** mRNA levels in DRG of lumbar (L4-L5) and cervical (C6-C8) spinal segments of removed from ipsilateral (i) and contralateral (c) sides of Naïve as well as sham-, SNC- and CSNT-operated rats for 7 days; *n* = 6 for each group. Relative expressions were determined using Actin as the housekeeping gene and normalized to naïve controls. *Significant difference (*p* < 0.05) compared to sham-operated rats in a Mann-Whitney U-test.

Similar to the GAP-43 and SCG-10 protein results, RT-PCR revealed a substantially smaller increase in SCG-10 mRNA levels in lumbar DRG (only up to 2.6-fold of sham-operated controls) than was measured for GAP-43 mRNA. Levels of SCG-10 mRNA were higher bilaterally in lumbar DRG after CSNT than after SNC, but the differences were not statistically significant. Both SNC and CSNT induced an approximately similar elevation of SCG-10 mRNA in cervical DRG of both sides compared to naïve and sham-operated controls (Figure 5).

### Sciatic Nerve Lesion Induced Activation of cJun and p38 MAPK in Cervical DRG Neurons

**Figure 6:**
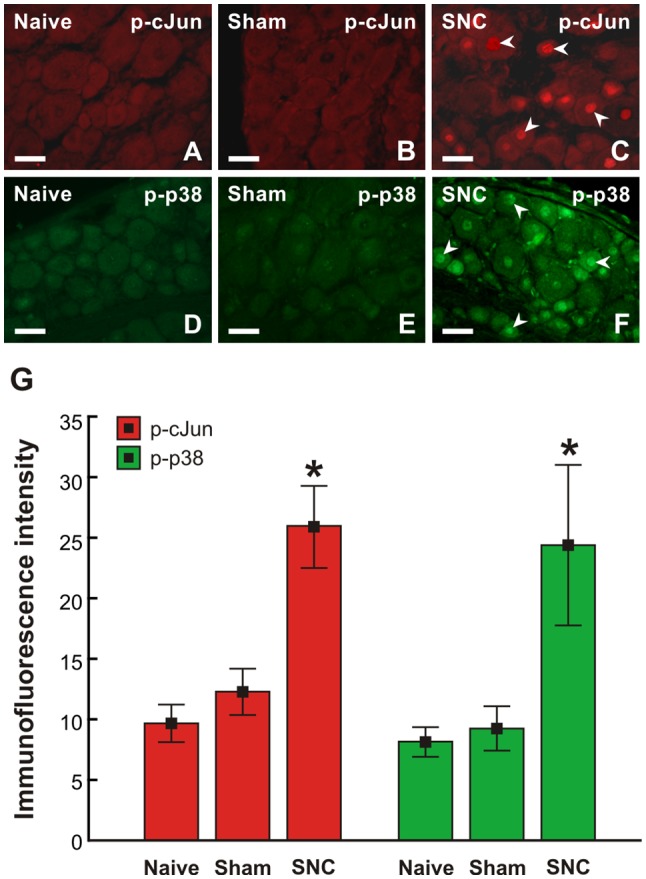
Representative pictures of cervical (C7) DRG neurons of intact rats **(A–D)** and rats 7 day after sham- **(B,E)** or SNC-operation **(C,F)**. DRG sections were immunostained for p-cJun **(A–C)** and p-p38 **(D–F)**. Scale bars = 40 μm.** (G)** Graph illustrating mean immunofluorescence intensity of activated p-cJun (red columns) and p-p38 (green columns) in cervical DRG neurons of naïve, sham, and SNC groups. SNC induced activation of p-cJun and p-p38 (arrowheads, **C,G**). *Significant difference (*p* < 0.05) compared to naïve or sham-operated rats in a Mann-Whitney U-test.

To confirm the sciatic nerve injury-induced pro-regenerative state of cervical DRG neurons demonstrated by GAP-43 and SCG10 analyses, activation of p-cJun and p-p38 MAPK was also investigated. Basal p-cJun and p-p38 MAPK immunofluorescence was very low in cervical DRG neurons of naïve and sham-operated rats. Sciatic nerve lesion increased immunofluorescence intensity of both p-cJun and p-p38 MAPK in the nuclei of cervical DRG neurons. Further, a higher intensity of p-p38 MAPK immunofluorescence was seen in the soma of neurons (Figure 6).

### Axon Regeneration Assay in Crushed Ulnar Nerve After Prior Sciatic Nerve Injury

**Figure 7:**
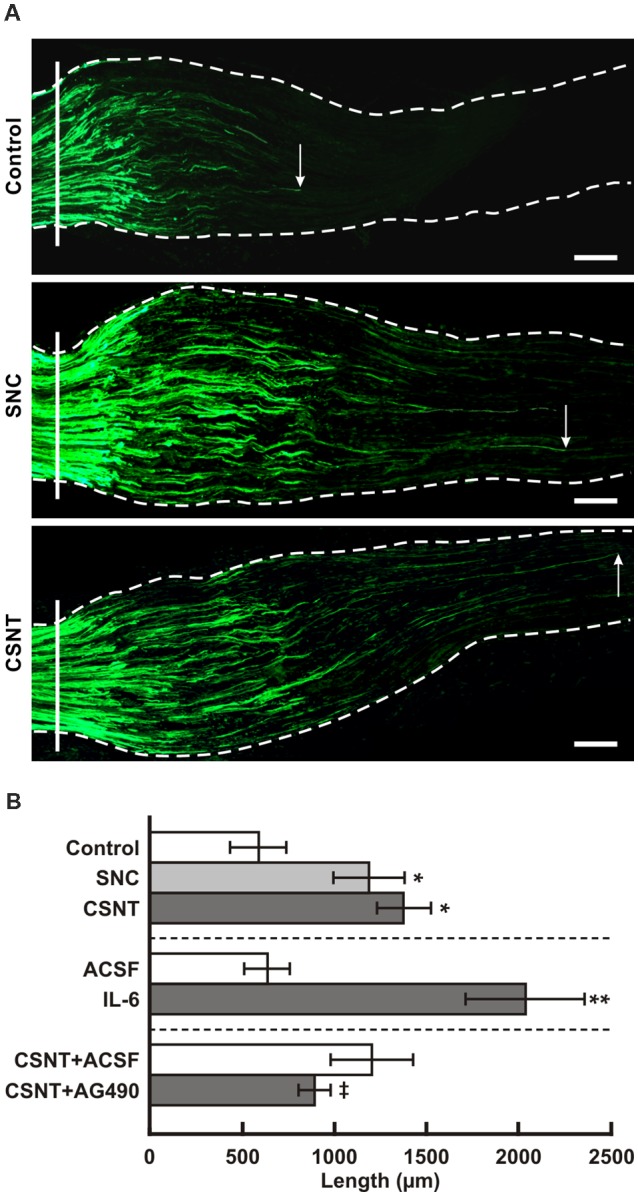
**(A)** Representative longitudinal sections through the ulnar nerves distal to the crush site (solid line) showing SCG-10 positive regenerated axons. Arrows indicate the tip of the longest SCG-10 positive axons. The ulnar nerves were removed after 1 day from control rat (only ulnar nerve crush) and from rats 7 days after prior SNC or CSNT. Scale bars = 100 μm. **(B)** The top portion of the graph illustrates mean length of regenerated SCG10+ axons ± SD in the ulnar nerve 1 day after crush in rats without a sciatic nerve injury (Control) and following 7 days from SNC or CSNT; *n* = 6 for each group. *Significant difference (*p* < 0.05) compared to control. The middle portion illustrates mean length of regenerated SCG10+ axons ± SD in the ulnar nerve 1 day after crush and intrathecal injection of 10 μl of artificial cerebrospinal fluid (ACSF) or IL-6 (20 ng/10 μl); *n* = 4 for each group. **Significant difference (*p* < 0.05) compared to control. The bottom portion illustrates mean length of regenerated SCG10+ axons ± SD in the ulnar nerve 1 day after crush 7 days from prior CSNT and intrathecal application of ACSF (CSNT+ACSF) or JAK2 inhibitor (CSNT+AG490); *n* = 4 for each group. ^‡^Significant difference (*p* < 0.05) compared to CSNT+ACSF group in a Mann-Whitney U-test.

The axonal regeneration capacity of cervical DRG neurons was investigated in longitudinal sections of one-day-old crushed ulnar nerves by immunostaining for SCG-10—a specific marker for regenerated axons of primary sensory neurons (Shin et al., 2014). Axon regeneration index expressed as SCG-10+ axon extension from the point of nerve crush was greater in SNC- and CSNT-operated rats compared to control rats with only ulnar nerve crush (Figures 7A,B).

### *In vitro* Assay of Axonal Outgrowth Capacity of Cervical DRG Neurons After Sciatic Nerve Injury

**Figure 8:**
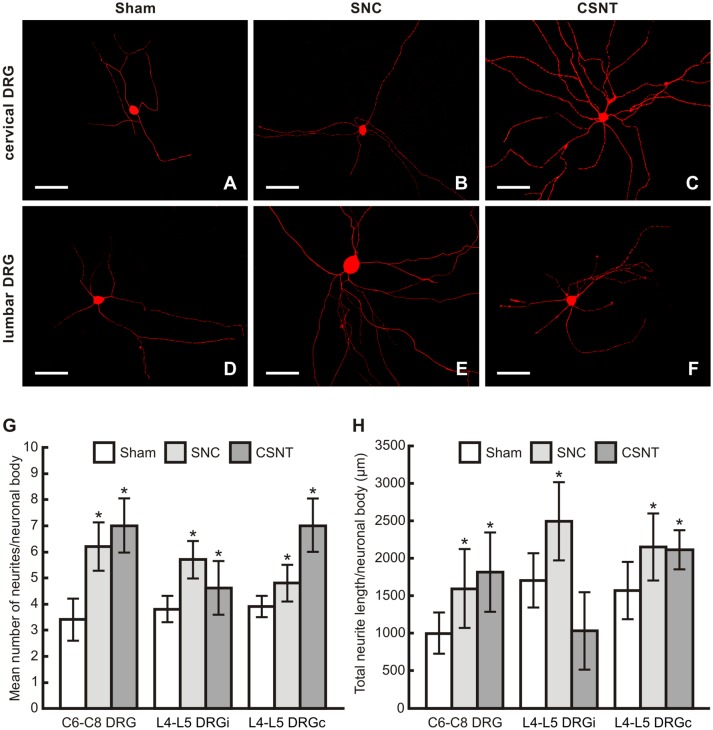
**(A–F)** Representative pictures of cervical (C6-C8) and lumbar (L4-L5) DRG neurons of the ipsilateral side dissociated and cultured *in vitro* after removing of DRG from sham-, SNC- or CSNT-operated rats for 7 days. The DRG neurons were fixed and immunostained for βIII-tubulin after 2 days of *in vitro* incubation. Scale bars = 75 μm. The graphs illustrate mean number of neurites **(G)** and total neurite length **(H)** per neuronal body of *in vitro* cultivated DRG neurons of cervical (C6-C8) and lumbar (L4-L5) spinal segments from the ipsilateral (i) and contralateral (c) sides removed from sham-, SNC- and CSNT-operated rats (*n* = 4 for each group). At least 100 randomly selected neurons with nuclei per cover slip were measured. *Significant difference (*p* < 0.05) compared to sham-operated controls in a Mann-Whitney U-test.

The increased capacity of cervical DRG neurons to regenerate their axons after prior sciatic nerve injury was verified by an *in vitro* assay of neurite outgrowth in DRG neurons taken from sham-, SNC- and CSNT-operated rats (Figures 8A–F). The diameter of neuronal bodies cultivated *in vitro* was between 25 and 45 μm, thus encompassing all morphological classes of DRG neurons.

The mean number of neurites per neuronal body of cervical and lumbar DRG taken from sham-operated rats was very similar. Compared to cervical DRG neurons of sham-operated rats, the number of neurites sent off by cervical DRG neurons taken from SNC- and CSNT-operated rats was significantly higher. Surprisingly, cervical and contralateral lumbar DRG neurons displayed higher neurite outgrowth than lumbar DRG neurons ipsilateral to CSNT (7.0 ± 2.1 and 7.0 ± 1.7 compared to 4.6 ± 1.5; Figure 8G).

The total length of neurites per neuron was significantly larger in neurons from lumbar DRG of both sides and those cultivated from cervical DRG 7 days after SNC. Seven days after CSNT, the total neurite length per neuron was significantly larger in neurons cultivated from cervical and contralateral lumbar DRG, but not in neurons cultivated from ipsilateral lumbar DRG (Figure 8H).

### IL-6 Protein Level in Plasma

**Figure 9:**
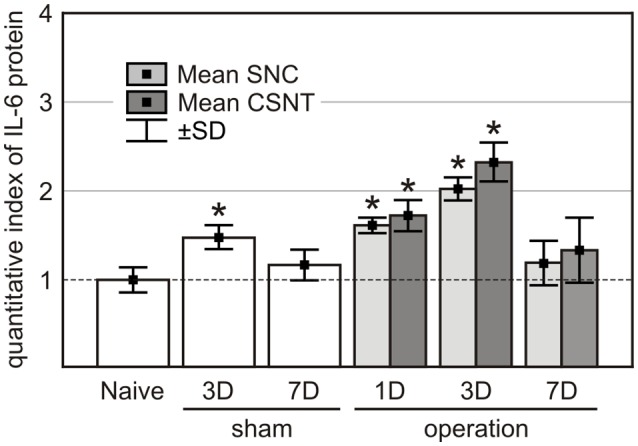
IL-6 protein levels in the plasma of naïve, sham-, SNC- and CSNT-operated rats (*n* = 3 for each group). Blood samples of sham-operated rats were obtained 3 (3D) and 7 (7D) days after surgical treatment and those from SNC- and CSNT-operated rats were obtained 1 (1D), 3 (3D), and 7 (7D) days after operation. *Significant difference (*P* < 0.05) compared to baseline levels of naive rats in a Mann-Whitney U-test.

IL-6 protein levels increased significantly in the plasma of sham-operated rats at 3 days but returned to normal by 7 days after treatment. Plasma IL-6 levels were elevated also in SNC and CSNT rats at 1 and 3 days but dropped back close to normal 7 days after sciatic nerve lesions (Figure 9). These IL-6 plasma level measurements are consistent with our previous results following SNC (Dubový et al., 2013) and indicate that IL-6 in the blood is likely not inducing the pro-regenerative state in cervical DRG neurons 7 days after sciatic nerve lesion.

### Axon Regeneration Assay in Crushed Ulnar Nerve and Changes in the Pro-regenerative State in Cervical DRG Neurons After Intrathecal Application of IL-6 and JAK2 Inhibitor

**Figure 10:**
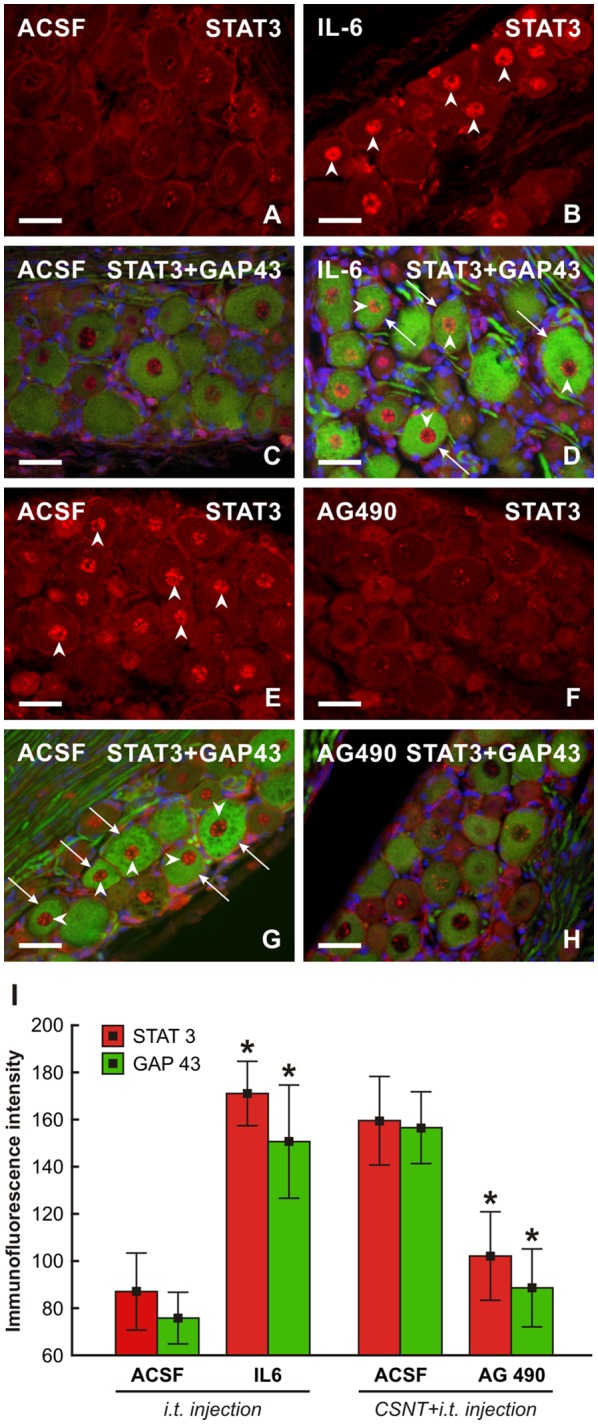
Representative pictures of cervical (C7) DRG neurons of intact rats **(A–D)** and rats 1 day after the ulnar nerve crush following 7 days from CSNT **(E–H)**. In intact rats, 10 μl of ACSF **(A,C)** or IL-6 **(B,D)** were intrathecally applied for 1 day. Cervical DRG were also removed from rats with CSNT for 7 days, a subsequent ulnar nerve crush for 1 day and intrathecal injection of 10 μl of ACSF **(E,G)** or AG490 **(F,H)**. DRG sections were immunostained for STAT3 **(A,B,E,F)** or double immunostained **(C,D,G,H)** for STAT3 (red fluorescence) and GAP-43 (green fluorescence). Intrathecal injection of IL-6 induced activation and nuclear translocation of STAT3 (arrowheads, **B,D**) as well as increased immunostaining for GAP-43 in neurons (arrows, **D**) when compared with ACSF treatment **(C)**. Cervical DRG neurons of rats following the ulnar nerve crush 7 days after CSNT displayed activation and nuclear translocation of STAT3 **(E,G)** and intense GAP-43 immunofluorescence in the neurons when injected with ACSF (**G**, arrowheads and arrows, respectively) while intrathecal application of AG490 resulted in a marked reduction of STAT3 activation and GAP-43 immunostaining **(F,H)**. Scale bars = 40 μm. **(I)** Graph illustrating mean immunofluorescence intensity of activated STAT3 and GAP-43 in cervical DRG neurons of intact rats following intrathecal application of ACSF or IL-6 for 1 day and rats with CSNT for 7 days and intrathecal injection of ACSF or AG490. *Significant difference (*P* < 0.05) compared with ACSF-treated rats in a Mann-Whitney U-test.

To investigate if IL-6 is responsible for activation of the pro-regenerative state of rat cervical DRG neurons, we intrathecally applied IL-6. We showed previously that intrathecal application of IL-6 induced activation and nuclear translocation of STAT3 in DRG neurons (Dubový et al., 2018a). In the present experiments, we observed that intrathecal injection of IL-6 increased not only the activation of STAT3 but also the expression of GAP-43 in intact DRG neurons. In contrast, application of AG490 after CSNT and ulnar nerve crush resulted in decreased STAT3 activation and expression of GAP-43 in cervical DRG (Figure 10).

In addition, we measured the lengths of SCG-10+ regenerated axons distal to the ulnar nerve crush following intrathecal IL-6 application. The regenerated axons were significantly longer compared to control ulnar nerve crush or nerve crush and subsequent intrathecal injection of ACSF (Figure 7B, the middle portion). The pro-regenerative state of neurons is mediated by phosphorylation of STAT3 at the Y705 position by JAK2 (Schwaiger et al., 2000; Qiu et al., 2005; Niemi et al., 2016). When an inhibitor of JAK2 (AG490) was applied intrathecally in rats with ulnar nerve crush for a day following prior CSNT, SCG-10+ regenerated axons were significantly shorter compared to those of the rats subjected to CSNT and subsequent ulnar nerve crush and intrathecal injection only of ACSF (Figure 7B, the bottom portion).

## Discussion

Activation of the neuronal regenerative program after axon injury is an important prerequisite for useful reinnervation and functional recovery. This pro-regenerative status of the neurons is linked with the upregulation of regeneration-associated molecules (Ma and Willis, 2015). For example, regeneration-associated proteins like GAP-43 and SCG-10 are frequently used as molecular markers of the pro-regenerative state of neurons induced by experimental nerve injury. The DRG neurons are a useful model for studying the induction of the regeneration-associated program marked by increased levels of GAP-43 and SCG-10 mRNA and protein (Mason et al., 2002; Shin et al., 2014). The DRG neurons of naïve rats display a low basal level of GAP-43 or SCG-10 immunostaining that is significantly increased in the neuronal bodies over a long period after sciatic nerve injury (Stewart et al., 1992; Schreyer and Skene, 1993; Shin et al., 2014; Dubový et al., 2018b).

GAP-43 immunostaining is a widely used marker for regenerating axons in experimental models of peripheral nerve injury. Apart from regenerating axons, intense GAP-43 immunoreactivity is also present in Schwann and Schwann-derived cells (Plantinga et al., 1993; Dubovy and Aldskogius, 1996). This GAP-43 immunostaining of Schwann cells associated with regenerated axons makes their detection difficult, especially during early periods of axon regeneration (Dubový et al., 2018b). In contrast to GAP-43, SCG-10 is transported from the soma to the proximal axonal stump very early after axotomy, whereas it is rapidly lost in distal axon stumps (Tararuk et al., 2006; Shin et al., 2012, 2014). In contrast to GAP-43, which labels regenerated axons after 3 days (Sar Shalom and Yaron, 2014), SCG-10 immunostaining is rapidly increased in proximal axonal stumps and regenerating sprouts within hours after axotomy (Shin et al., 2012, 2014). Moreover, SCG-10 is a marker for regenerated sensory axons of injured peripheral nerves (Shin et al., 2014). Therefore, we monitored GAP-43 and SCG-10 mRNA and protein to prove the pro-regenerative state of DRG neurons, and SCG-10 was used to assess axon regeneration distal to ulnar nerve crush after 1 day.

### Unilateral Sciatic Nerve Lesion Induced Pro-regenerative State in Both Lumbar and Cervical DRG

Subpopulations of rat DRG neurons can be classified according their size, their cytological, chemical and physiological properties (Lawson, 2002) and behave differently with respect to their regeneration capacity as well as the expression of molecular regeneration markers (Bonilla et al., 2002; Leclere et al., 2007; Shin and Cho, 2017). Classically, DRG neurons are divided into large-, medium- and small-sized (Lawson et al., 1974) and may also be identified by further molecular phenotyping. Quantitative immunohistochemical staining revealed increased GAP-43 and SCG-10 immunofluorescence in both lumbar and cervical DRG neurons ipsilateral as well as contralateral to the SNC or CSNT. Detailed image analysis showed that the predominant increase of GAP-43 immunofluorescence intensity was seen in the large-sized neuronal subpopulation of both lumbar and cervical DRG. In contrast, a similar elevation of SCG-10 immunofluorescence intensity was observed in all size-classes neurons of DRG in the lumbar and cervical spinal cord segments. Principally, bilateral increase of GAP-43 and SCG-10 proteins detected by quantitative immunohistochemical staining in both lumbar and cervical DRG neurons after sciatic nerve lesion was verified by measuring whole GAP-43 and SCG-10 protein levels using western blot analysis. Further, it also revealed that unilateral sciatic nerve lesion induced bilateral elevation of activated (phosphorylated) GAP-43 in both lumbar and cervical DRG.

The pro-regenerative state of DRG neurons in both lumbar and cervical spinal cord segments was confirmed by increased levels of GAP-43 and SCG-10 mRNA. Although SCG-10 is considered a better marker of the pro-regenerative state of DRG neurons (Shin et al., 2014; Dubový et al., 2018b), increases in the levels of GAP-43 mRNA and protein were more distinct compared to naïve or sham-operated rats in both lumbar and cervical DRG after SNC or CSNT for 7 days. Our observation of a bilateral increase in GAP-43 and SCG-10 mRNA and proteins correlates well with published results indicating that NGF mRNA is bilaterally increased in both cervical and lumbar DRG after unilateral sciatic nerve crush (Heumann et al., 1987; Wells et al., 1994). These results suggest that upregulation of growth-associated molecules is not restricted to axotomized DRG neurons and probably reflects a systemic response of DRG neurons to unilateral sciatic nerve injury (Dubový et al., 2013, 2018a).

The expression of regeneration-associated proteins like GAP-43 or SCG10 is operated by transcription factors, such as c-Jun (Raivich et al., 2004; Frey et al., 2015; Valakh et al., 2015) or members of the MAPK family, e.g., p38 (Verma et al., 2005; Temporin et al., 2008; Nix et al., 2011; Law et al., 2016). Our results showing increased levels of GAP-43 and SCG10 as well as activation of p-cJun and p-p38 in cervical DRG after sciatic nerve injury suggested that the pro-regenerative program can be induced in remote cervical DRG neurons 7 days after sciatic nerve lesion.

### Increased *in vivo* and *in vitro* Axon Regeneration Capacity of Cervical DRG Neurons Confirmed Their Pro-regenerative State Induced by Prior Sciatic Nerve Lesion

While SCG-10 increases only in the proximal stumps of afferent axons (Shin et al., 2014), we also measured SCG-10 in regenerated peripheral arms of afferent axons associated with cervical DRG neurons distal to the ulnar nerve crush. SCG-10 immunopositive axons displayed a significantly greater length from the point of ulnar nerve crush after prior SNC or CSNT than in controls without the conditioning lesion. These results demonstrated *in vivo* the enhanced pro-regenerative state of cervical DRG neurons corresponding with increased levels of GAP-43 or SCG-10 induced by the conditioning sciatic nerve lesion.

Neurite outgrowth assays are used to assess the effects of *in vivo* manipulations performed prior to removing DRG from animals (Frey et al., 2015; Al-Ali et al., 2017). In our *in vitro* assay, the number of neurites and their total lengths per neuron were significantly higher in cervical DRG neurons removed from rats subjected to SNC or CSNT than those from sham-operated controls. The results of the *in vitro* assay confirmed the enhanced capacity of cervical DRG neurons to regenerate their neurites after the conditioning sciatic nerve lesion.

Thus, the axon outgrowth tested *in vivo* and *in vitro* proves the initiation of pro-regenerative state in cervical DRG neurons non-associated with sciatic nerve lesion. Our present results extend previously published results that unilateral nerve injury affects the uninjured contralateral nerve with respect to expression of inflammatory mediators (Ruohonen et al., 2002) including *in vivo* promotion of axonal regeneration in the contralateral nerve associated with enhanced cytokine expression in the contralateral DRG (Ryoke et al., 2000).

### IL-6 Is a Candidate for Signaling the Pro-regenerative Neuronal State in Remote DRG After Sciatic Nerve Lesion

In response to unilateral sciatic nerve injury, mRNA and protein levels of IL-6 and its receptors were enhanced bilaterally in primary sensory neurons not only in DRG of lumbar segments (L4-L5) associated with the injury, but also in cervical segments (C7-C8) not associated with the injured nerve (Brázda et al., 2013; Dubový et al., 2013). Upon binding to its membrane-bound receptor (IL-6R), IL-6 connects the intracellular regions of gp130 to initiate a signal transduction cascade by activating signal transducer and activator of transcription 3 (STAT3; Eulenfeld et al., 2012). Activation of STAT3 by JAK2-dependent phosphorylation at the tyrosine-705 (Y705) position occurs in DRG neurons after a nerve lesion and is mediated by neuropoietic cytokines including IL-6 (Schwaiger et al., 2000; Sheu et al., 2000; Qiu et al., 2005; Miao et al., 2006) and neurotrophins (Ng et al., 2006; Pellegrino and Habecker, 2013). We found STAT3 activation and nuclear translocation bilaterally in the DRG neurons of both lumbar and cervical spinal cord segments after unilateral SNC or CSNT. Moreover, we also proved increased levels of IL-6 protein in the CSF following nerve injury as well as activation and nuclear translocation of STAT3 in DRG neurons along the neuroaxis after intrathecal injection of IL-6 (Dubový et al., 2018a). It is known that activation of STAT3 by JAK2 phosphorylation is a prerequisite for the synthesis of regeneration-associated proteins like GAP-43 or SCG-10 (Patodia and Raivich, 2012) and axon regeneration (Bareyre et al., 2011; Niemi et al., 2016). Intrathecal application of IL-6 in our present experiments increased STAT3 activation and GAP-43 expression in cervical DRG neurons, while a JAK2 inhibitor (AG490) decreased STAT3 activation and nuclear translocation as well as expression of GAP-43.

Intrathecal delivery of IL-6 promotes regeneration of the central arms of afferent axons into the spinal dorsal cord. This then activates the neuronal regeneration program in DRG neurons to overcome inhibitors of axon regeneration present in myelin (Cao et al., 2006). Therefore, in our experiments we tested *in vivo* the effect of intrathecal IL-6 and JAK2 inhibitor injection on axon regeneration after an ulnar nerve crush to illustrate the pro-regenerative state of cervical DRG neurons. Intrathecal IL-6 injection significantly increased the lengths of SCG-10+ regenerated axons distal to the ulnar nerve crush. In contrast, intrathecal application of AG490 reduced the lengths of regenerated axons after the ulnar nerve crush following prior unilateral SNC or CSNT. These results suggested a critical role for the JAK/STAT signaling pathway activated by IL-6 in inducing the pro-regenerative state in remote DRG neurons after unilateral sciatic nerve lesion.

What remains unclear is the mode of activation of remote DRG neurons following a conditioning unilateral sciatic nerve lesion. Axonal transport of local WD signaling molecules would be implicated only on the ipsilateral side of the compressed nerve (SNC) but can be excluded when the similar bilateral changes were found in CSNT group of rats. We observe bilateral changes in DRG of both lumbar and cervical segments, and this rather points towards a systemic pathway through either the blood stream or the CSF. The low level of IL-6 in the plasma of rats 7 days after SNC or CSNT suggests that it is more likely to be via the CSF. We found previously that rat DRG have no barrier to the CSF of the spinal subarachnoid space (Joukal et al., 2016). Therefore, intrathecal injection of IL-6 with subsequent activation of STAT3 in cervical DRG neurons (Dubový et al., 2018a) can trigger upregulation of GAP-43 and SCG-10 associated with the pro-regenerative state of these neurons. A unilateral sciatic nerve lesion induces increased IL-6 synthesis in the corresponding lumbar DRG (Murphy et al., 1995; Dubový et al., 2013) and the cytokine molecules can be released into the subarachnoid space and transported via CSF into the remote DRG to activate STAT3 (Dubový et al., 2018a) and this then induces the neuronal pro-regenerative state.

Finally, our results indicate that the pro-regenerative state of cervical DRG neurons illustrates a systemic reaction along the neuroaxis to unilateral sciatic nerve injury, and that this reaction can be mediated by IL-6 and JAK2/STAT3 signaling. Moreover, the results suggest a role for inflammatory mediators in activating the neuronal pro-regenerative state without direct retrograde axonal transport of signaling molecules from the injured nerve.

## Conclusion

In the present study, we have demonstrated that sciatic nerve lesion for 7 days triggers bilateral activation of a pro-regenerative state not only in the primary sensory neurons of lumbar DRG (L4-L5) associated with injured nerve but also in remote cervical DRG. The bilateral activation of the pro-regenerative state in DRG neurons anatomically non-associated with the injured nerve correlates well with our previous observation of bilateral expression of IL-6 and activation of STAT3. Taken together, these results indicate that the systemic reaction of DRG neurons to a unilateral nerve lesion along the neuroaxis can be mediated by IL-6 and JAK2/STAT3 signaling. This phenomenon can be activated also by other molecules released into the CSF of the perispinal subarachnoid space by neurons and non-neuronal cells in DRG associated with the injured nerve.

## Author Contributions

PD designed the research and wrote the article. IK, IH-S and MJ performed *in vivo* experiments and prepared samples for immunohistochemistry, western blot and RT-PCR and wrote the corresponding texts. MK performed *in vitro* experiments and prepared samples for immunohistochemistry and wrote the corresponding text. VB performed and analyzed western blot and RT-PCR data and wrote the corresponding text.

## Conflict of Interest Statement

The authors declare that the research was conducted in the absence of any commercial or financial relationships that could be construed as a potential conflict of interest.
